# The CATFISH study protocol: an evaluation of a water fluoridation scheme

**DOI:** 10.1186/s12903-016-0169-0

**Published:** 2016-02-01

**Authors:** Michaela Goodwin, Richard Emsley, Michael Kelly, Eric Rooney, Matthew Sutton, Martin Tickle, Rebecca Wagstaff, Tanya Walsh, William Whittaker, Iain A Pretty

**Affiliations:** The Dental Health Unit, School of Dentistry, The University of Manchester, Williams House, Manchester Science Park, Manchester, M15 6SE UK; Centre for Biostatistics, Institute of Population Health, The University of Manchester, Manchester Academic Health Science Centre, 1.304 Jean McFarlane Building, Oxford Road, Manchester, M13 9PL UK; Institute of Public Health, University of Cambridge, Forvie Site, Cambridge, CB2 OSR UK; Public Health England, North West, 1st Floor, York House, Ackhurst Business Park, Foxhole Road, Chorley, PR7 1NY UK; Jean McFarlane Building, Oxford Road, Manchester, M13 9PL UK; Coupland 3 Building, Oxford Road, Manchester, M15 6FH UK; Dental Observatory, NHS Central Lancashire, Preston Business Centre, Watling Street Road, Fulwood, PR2 8DY UK

## Abstract

**Background:**

Tooth decay is the commonest disease of childhood. We have known for over 90 years that fluoride can prevent tooth decay; it is present in nearly all toothpastes and can be provided in mouthwashes, gels and varnishes. The oldest method of applying fluoride is via the water supply at a concentration of 1 part per million. The two most important reviews of water fluoridation in the United Kingdom (the York Review and MRC Report on water fluoridation and health) concluded that whilst there was evidence to suggest water fluoridation provided a benefit in caries reduction, there was a need to improve the evidence base in several areas.

**Methods/Design:**

This study will use a natural experiment to assess the incidence of caries in two geographical areas, one in which the water supply is returned to being fluoridated following a discontinuation of fluoridation and one that continues to have a non-fluoridated water supply. The oral health of two discrete study populations will be evaluated - those born 9 months after the water fluoridation was introduced, and those who were in their 1st year of school after the introduction of fluoridated water. Both populations will be followed prospectively for 5 years using a census approach in the exposed group along with matched numbers recruitment in a non-exposed control. Parents of the younger cohort will complete questionnaires every 6 months with child clinical examination at ages 3 and 5, whilst the older cohort will have clinical examinations only, at approximately 5, 7 and 11 years old.

**Discussion:**

This project provides a unique opportunity to conduct a high quality evaluation of the reintroduction of a water fluoridation scheme, which satisfies the inclusion criteria stipulated by the York systematic review and can address the design issues identified in the MRC report. The research will make a major contribution to the understanding of the costs and effects of water fluoridation in the UK in the 21st Century. Its findings will help inform UK policy on this important public health intervention and may have a significant impact on public health policy in other developed countries. There is currently true equipoise in relation to the effectiveness of water fluoridation in contemporary populations and while the biological plausibility is well established, there is a need to examine impact on the changing epidemiological status of dental decay.

## Background

Dental caries remains a significant public health problem and is certainly one of the most prevalent diseases affecting children [1–3]. The most recent UK national survey [4] reported that 43 % of 5-year-olds had tooth decay. The prevalence of tooth decay varied between countries, from 41 % in England to 52 % in Wales and 61 % in Northern Ireland (data for Scotland were not reported). The survey showed little change since the 1993 national survey [5], which reported a prevalence of 45 %. More recent data from NHS surveys showed little sign of improvement [6].

Disease in permanent teeth has fallen rapidly over the last 30 years; with the prevalence of obvious decay experience in 12 years olds in England falling from 81 % in 1983, to 52 % in 1993 and 34 % in 2003 [4]. This picture of overall improvement in population prevalence masks significant social inequalities in tooth decay. In addition national surveys do not report disease statistics among vulnerable groups. The costs to the NHS of treating tooth decay are very significant. In England alone the NHS dental allocation in 2011–12 was £2.3 billion, this is net of patient charges, which roughly makes up a quarter of the total budget, and does not include the budgets for community and hospital services or the costs of care provided by the private sector. The majority of this funding is to pay for the detection and treatment of dental caries. While this budget includes both adults and children it important to consider the fact caries can be a lifelong disease. When a permanent tooth is restored this can commence a life long treatment and retreatment cycle [7, 8].

Tooth decay is strongly associated with poverty. Young children from poor families carry a disproportionate amount of the population disease burden [9, 10]. A UK prospective cohort study of 3–6-year-olds [11] showed that once a child develops the disease it progresses rapidly. It also has a significant impact; children with caries have a 25 % risk of experiencing pain and an 11 % risk of having an extraction each year [12]. If the disease is unchecked multiple extractions under general anaesthetic (GA) are the norm. Dental extractions are the commonest reason why young children have a GA. Exact figures are not available [13] but recent national guidance [14] estimated between 60,000 and 100,000 cases are carried out each year. We know GA extractions have a significant negative impact on young children and their families [15] and that there is a strong association between dental extractions and dental anxiety, which can continue to affect individuals in later life [16]. There are also significant inequalities in access and utilisation of dental services, with those with the greatest need least likely to access dental services [17]. This situation gives cause for concern, even more so, when the main disease the service is concerned with is wholly preventable by limiting sugar intake and adopting a rigorous self-care regime, which includes optimal use of topical fluorides. However, water fluoridation (WF) may mitigate behavioural differences in self-care and sugar intake that impact on dental caries and may be correlated with social class.

It has been known for over 90 years that fluoride can prevent tooth decay and it has been noted that the improvement in oral health seen over the past 30 years is attributed mainly to the introduction of fluoride on a mass scale [18]. The oldest method of applying fluoride is via the water supply at a concentration of 1 part per million. Early trials of water fluoridation in the USA and UK in the 40s and 50s showed very dramatic falls in tooth decay [19]. However since the introduction of fluoride toothpaste in the 1970s there has been a significant fall in tooth decay. Public debate on water fluoridation tends to be highly polarised with very strong views held by the pro and anti lobbies. Unfortunately unequivocal trial and cohort based scientific evidence to tell us how well water fluoridation works and how cost effective it is in the current climate of reduced decay levels is lacking.

The two most important reviews of water fluoridation in the United Kingdom have been the York Review [20] and the MRC Report on water fluoridation and health [21]. The main conclusions of these reviews was that whilst there was evidence to suggest water fluoridation provided a benefit in caries reduction, there was a need to improve the evidence base in several areas:

- *A recommendation that fluoride exposure in children should be explored against a background of exposure to other sources of fluoride, particularly toothpaste*
- *Greater knowledge on how social class affects fluorosis risk, linked to the differences in caries experience between social classes*
- *Researchers needed to address issues surrounding bias in caries and fluorosis examinations, with consideration given to blinding of assessments and a more objective approach to assessments.*

There is a unique opportunity to study the impact of water fluoridation in West Cumbria using a natural experiment. A water fluoridation scheme was established in the 1960s but has been off line for several years; the plant came back on line in 2013. This CATFISH project (Cumbrian Assessment of Teeth a Fluoride Intervention Study for Health) aims to provide strong evidence of the effects and costs of a ‘reintroduced’ water fluoridation scheme on young children.

The study objectives are:

- To assess the effects and costs of both systemic (exposure from in utero) and topical exposure to water fluoridation following the introduction of a WF scheme on a contemporary birth cohort of children, as compared to a birth cohort of children not exposed to WF.
- To assess the effects and costs of topical exposure (exposure from approximately 5 years old onwards -those who are in their first year of school) to water fluoridation alone following the introduction of a WF scheme on a cohort of contemporary children (falling disease levels), as compared to a cohort of children in the absence of WF.
- To measure the impact of water fluoridation on social class inequalities in child dental health.
- Using a research design that meets the requirements of a new scheme evaluation described by both the York CRD and MRC reviews.
- To assess the cost-effectiveness of a WF scheme.

## Methods/Design

This is a prospective, comparative, population based study across two age groups (both birth and 5 year olds age groups), to examine the effects of the reintroduction of water fluoridation, on young children’s oral and general health. The prospective study will recruit two distinct age groups. One will examine children who will be in their first year of school in 2013 (further referred to as the topical only group or *TO*) and the second group will follow children born the following year starting from September 2014 as those in the exposed group will have received water fluoridation from conception (further referred to as the systematic and topical group or *S&T*) (see Table 1).

**Table 1:** Information for both age groups involved in the study

Group	Sample /Design	Recruitment begins	Data collection begins	School Year	Age child will be at start the study
Topical only	Census/ Matched group	September 2013	September 2013	1st year of school in 2013	4/5 years old
Systematic and topical	Census	May 2014	September 2014	1st year of school in 2019	From birth

S&T children will be followed prospectively using a census approach over the next 5 years of their life, with survey, clinical and environmental data collected (Table 2). Baseline data will be collected from new mothers and fathers (primary carers) shortly after the birth of their child, and data will be collected about primary carers and children every 6 months from birth until the child is 5 years old. TO children will be followed until they reach 11 with clinical examinations at ages 5, 7 and 11 and questionnaires given out in the first year of data collection (Table 3). This TO cohort will use a census approach in the exposed population (covering the West of Cumbria) and a comparable group in the non-exposed population across the North of Cumbria.

**Table 2:** Study timeline and data collection for S&T group

Measures	Child Age										
Age in Years	0		1		2		3		4		5
Age in Months	0	6	12	18	24	30	36	42	48	54	60
Child dental examination							✓				✓
Household environment & demographics	✓	✓	✓	✓	✓	✓	✓	✓	✓	✓	✓
CHILD											
Oral Hygiene Behaviours		✓	✓	✓	✓	✓	✓	✓	✓	✓	✓
Clinical examination							✓				✓
Child general health	✓	✓	✓	✓	✓	✓	✓	✓	✓	✓	✓
Chu9D											✓
Diet - weaning practices	✓	✓	✓	✓	✓	✓	✓	✓	✓	✓	✓
Body Mass Index	✓	✓	✓	✓	✓	✓	✓	✓	✓	✓	✓
Access to fluoride	✓	✓	✓	✓	✓	✓	✓	✓	✓	✓	✓
Access to dental treatment/ services		✓	✓	✓	✓	✓	✓	✓	✓	✓	✓
Serious Adverse Events	✓	✓	✓	✓	✓	✓	✓	✓	✓	✓	✓
Occurrence of dental pain		✓	✓	✓	✓	✓	✓	✓	✓	✓	✓
Hospital visits for dental		✓	✓	✓	✓	✓	✓	✓	✓	✓	✓
General Anaesthetic Extraction											✓
PARENT											
Oral Hygiene Behaviours	✓										✓
Self-reported oral health status	✓										✓
Fluoride Levels (Household Water Supply)	✓										✓
Attitudes and Choice (water consumption)	✓										✓
Dental visits	✓										✓

**Table 3:** Study timeline and data collection for TO group

Measures	Child Age						
Age in Years	5	6	7	8	9	10	11
Child dental examination	✓		✓				✓
Chu9D	✓						✓
Oral Hygiene Behaviours	✓		✓				✓

## Participants and recruitment

**Fig. 1:**
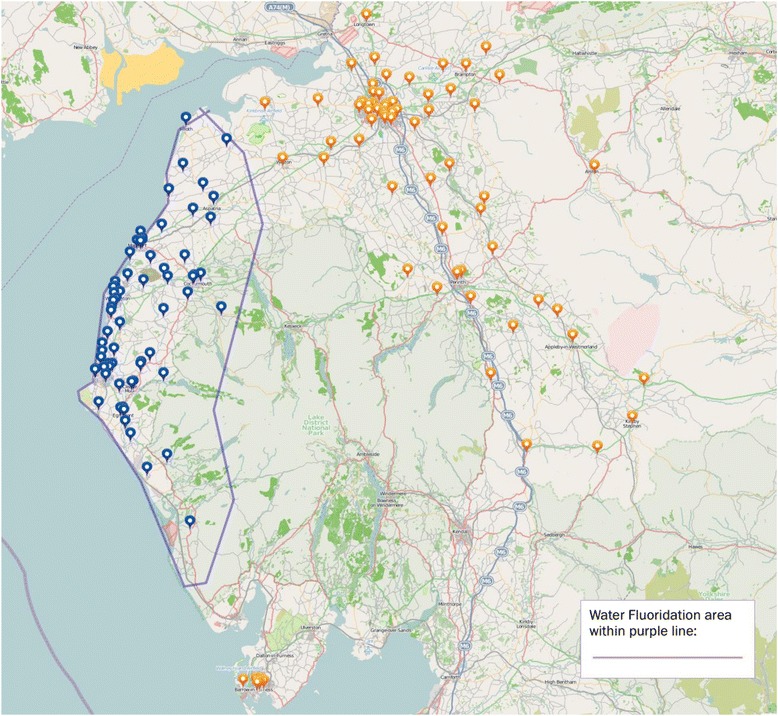
Map of recruitment and water fluoridation in Cumbria

Participants will be recruited from across Cumbria (see Fig. 1 for a map of Cumbria with area of recruitment by school and water fluoridation defined). While a population-based approach is taken (meaning there are potentially 3000 eligible children for recruitment) the minimum sample size required is 1044. This is based on the proportion of children who develop caries when ‘non-exposed’ to fluoride is 0.47 and the proportion when ‘exposed’ is 0.37. For a study such as this to be adequately powered to detect a risk difference of 0.1 (Risk Ratio 0.8) at 0.05 level with 90 % power a total sample size of 1044 children would be required which is well within our anticipated recruitment capabilities.

*S&T:* The birth cohort will be recruited from two sites – the exposed population (West Cumbria) and a control population (non fluoridated areas of Cumbria). New parents will be recruited during pregnancy and post-natally (examples include; 20 week scan, after delivery and at health visits within the first 3 months of birth). Parents of all children who are born at the two hospitals within Cumbria will be approached. Given the planned census approach, we will approach up to 3200 new parents and retain the parent–child dyads over this five-year study. With an anticipated consent rate of 84 % (based on previous experience in this population from the NHS Dental Epidemiological Surveys [22]), assuming refusal of dental exam at 7.5 % and loss to follow up at 12.5 %, those available for the second clinical exam will be 1720. Due to the population based nature of the study and the potential to benefit at a population level the study has broad inclusion criteria with only those with significant health issues not eligible for inclusion in the study.

**Fig. 2:**
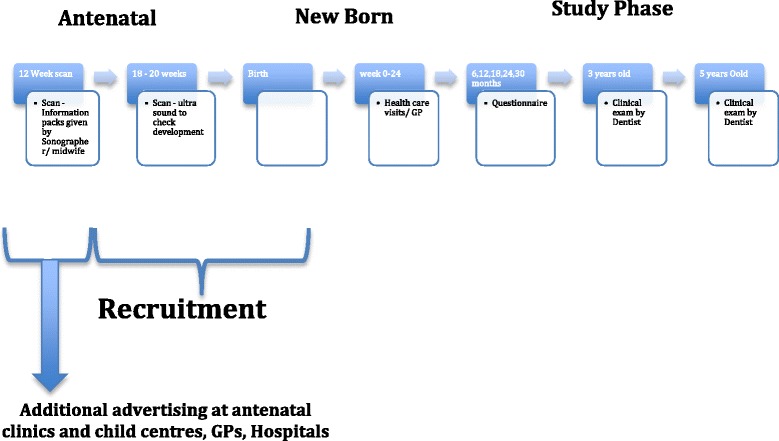
Recruitment outline for Birth Cohort

Exclusion criteria includes those individuals who are planning to move from the area within the duration of the study and those who are unable or unwilling to provide consent. Parents who agree to participate will provide written consent, complete a baseline questionnaire and be contacted again approximately every 6 months until their child reaches the age of 5. At age 3 and 5 a clinical exam carried out by a trained dentist will be performed (Fig. 2).

*TO* – The study population will be comprised of 5 year olds recruited from primary schools in the West of Cumbria (fluoridated) and a comparable group in the non fluoridated area of Cumbria. Similarly to the S&T group the parents of 5 year old children in the schools selected for the TO group will also be approached over one year and children will be followed through until they are 11 (in their last year of primary school). Inclusion criteria will be broad with an emphasis on capturing whole population effect. Consent will be obtained using parental written consent with child assent gained at time of clinical / dental examination. Children will only be excluded from participating if they are unable or unwilling to consent. Consent will be re-visited on each successive examination (using an opt-out system after initial written consent is given).

## Measures and procedures

### Questionnaires

For the S&T group participants will be asked to complete questionnaires after the birth of their child and every 6 months after this until their child is 5 years old. Questionnaires will include items across several domains, such as socio-economic status and demographics, household water environment (source of water, use of filters, etc.), infant feeding practices, oral health care, use of general and oral health care services, dental treatments, and general and oral health status. Questionnaires will be tailored to the age of the child with a general questionnaire administered to parents when appropriate (see Table 1). For the TO group a shorter questionnaire on oral hygiene behaviours including brushing, toothpaste use and what the child has eaten an hour before bed will be asked during the first year of data collection. Questionnaires will be made available via post, online submission and also may be completed by telephone call.

#### Household data, demographics, water environment, attitudes and health status

These data will be collected via a written parental questionnaire at recruitment (after birth or the first health care visit when the child is born) and will include items to assess socio-economic status (SES) and demographics i.e.:

- Number of household members
- Parental education
- Parental occupation
- Household income

Equivalised household income will be measured using the McClements’ equivalence scale using data collected on gross household annual income and the number of adults and children resident at the child’s house. Equivalised household income will then be grouped into quintiles, to enable comparisons of the effects of WF.

The questionnaire will also contain items on water sources available to the household including water consumed for drinking.

Parents will also be asked to self-report their use of oral health care services, last dental treatment and general health and oral health status using the 5-point Likert scale used in the adult dental health survey [23] .

#### Health data

As well as reporting their own general and dental health, parents will also be asked about their child’s health and questions relating to; hospital visits, pain experienced, trouble sleeping, every 6 months throughout the 5 year study. A generic measure of the child’s health will be obtained using the Child Health Utility 9 Dimension (CHU9D) instrument.

Parents will also be asked at recruitment to provide consent for the study researchers to collect data from relevant medical (i.e. Red book- child health record) and dental records.

#### Anthropometry

Parents will be asked their child’s weight and length/height recorded at each 6-month interval. This information may also be gathered from doctors/ healthcare visitor visits.

#### Dietary measures

A recent study examining water fluoridation in areas over the North of England indicated that sugar before bed was an important predictive risk factor for adolescent caries. Therefore, this will be the main dietary measure to avoid lengthy diet diaries impacting on participant response fatigue. Additionally parents will be asked about diet and weaning practices with milk formula, juice or other in either a bottle or cup.

#### Fluoride exposure

Potential sources of fluoride are either through the fluoridated water in the exposed area and fluoride containing oral health products (e.g. toothpaste, rinses, gels).

- *Dietary sources of fluoride:* Information on fluoride exposure from dietary sources will be collected via questionnaires (including water use).
- *Water sources of fluoride:* Fluoride content of the water will be known from United Utilities from a monthly monitoring update.
- *Non-dietary sources of fluoride:* Measures of non-dietary ingestion will be estimated using pictorial information on how much toothpaste is used, frequency of brushing, product name (fluoride level) and age started using toothpaste will be recorded information will also be collected on use of fluoridated gels, tablets and varnishes.

Child questionnaires will be repeated every 6 months to assess variation in these measures across time, i.e. water consumption, health, dental visits (see Table 2).

#### Oral Health: dental examination

Children will undergo a dental examination when the child is 3 years old and 5 years old for the S&T study and 5, 7 and 11 for the TO study. Caries scores will be determined using BASCD criteria. Subject’s teeth will be cleaned and dried prior to being scored clinically by trained and calibrated assessors. Caries will be recorded if there are one or more carious lesions into dentine.

Intra-oral photographs taken with a SOPRO intra-oral camera will permit the remote, blinded scoring for caries. Images will be stored on a secure database prior to being scored [24, 25]. This method will provide comparison with the clinical caries scores, provide an archive of images and will facilitate longitudinal scoring of children. Examinations for the birth cohort children will be undertaken at 3, and 5 years of age. The prospective cohort of children will have caries examinations at baseline (5 years) and then again at 7 and 11 years (see Table 3). BASCD scoring will be used to calculate teeth with decay. The use of the BASCD system will permit calculation of thresholds of caries severity. The proportion of caries free individuals in each group will be calculated at each time point.

Fluorosis cannot be effectively measured at the ages the children will be in the study period. However this will be explored later if additional funding can be gained to continue to follow these children and examine any differences in fluorosis and associated factors.

## Analysis

### Health effects of water fluoridation

The primary objective is to determine whether there is a difference in the proportion of children in the fluoridated and non-fluoridated cohorts that develop one or more carious lesions into dentine during the period of observation (case definition is 1 or more carious lesions into dentine. This will be estimated by calculating the Incidence Rate Ratio/Risk Ratio between the exposed and unexposed groups. The’natural experiment’ in water fluoridation exposure implies an absence of confounders in this study (water fluoridation dosing in the exposed population is independent of social class, other fluoride sources etc.). If significant differences between the groups on key variables at baseline are found, we will perform regression analysis to adjust for these variables.

The statistical approach in the TO cohort will be identical. However, the first examination at year 5 will enable an early look at the baseline caries levels between the two populations to identify any baseline imbalance in caries that may require subsequent adjustment in the statistical analysis.

The results of the study will be reported in accordance with the STROBE guidelines for the reporting of observational studies [26].

We will consider the mediating role of changes in behavioral factors, such as fluoride use and change in diet, in explaining the relationship between WF and outcome. We will also examine potential effect modification of these measures at baseline, and of socio-economic status. In addition, the longitudinal nature of the study will provide a rich dataset on behaviours through which we can identify changes in oral health care and dietary habits in both cohorts, and model through multivariable regression the impact this may have, either positively or negatively, on the outcome. For instance, parents may place less importance on their child’s tooth brushing with fluoride toothpaste when they are receiving fluoridated water. Conversely, parents of children in the non-fluoridated cohort may engage more in caries preventive behaviour.

### Economic evaluation of water fluoridation

Cost-effectiveness will be assessed in accordance with NICE guidelines for technology appraisal [27]. The evaluation will assess the health effects of WF on children and the costs to the NHS and personal and social services.

The clinical outcome will be the number of caries. For the cost-effectiveness analysis, outcomes will be collected on child health-related quality of life (HRQOL) that can be transformed into utility scores in accordance with the NICE Reference Case. We will collect HRQOL via the CHU9D instrument. The CHU9D is a paediatric health related quality of life measure and has been validated for use in children aged 7–11 [28] and preference weights exist that have been derived from the UK population [29].

The HRQOL information will be completed by:

Older School Cohort (5 to 11 years old) will complete two questionnaires:

- Aged 5 - By parents during first year of school by postal / online questionnaire.
- Aged 11 - by children in school during examination

Birth Cohort (0–5 years old) will complete one questionnaire

- Aged 5 - By parents during first year of school by postal / online questionnaire.

Matching of the birth cohort to the older birth cohort at age 5 will enable the extrapolation of cost and utility figures for the birth cohort to age 11.

NICE guidance specifies that the time horizon needs to be long enough to capture all-important differences in costs and outcomes. The main analysis will consider the cost-effectiveness of water fluoridation within the trial period. This is a conservative assumption, as we expect benefits and cost savings in the short-term to continue into the long-term. Additional analysis will model the future impact of the changes in the primary outcome on future costs and benefits using estimates from the literature and analysis of cohort studies. We will explore the sensitivity of our results by exploring alternative scenarios when extrapolating the effects over the long-term.

The costs and outcomes will then be translated into incremental cost-effectiveness ratios. Uncertainty in the model will be accounted for via cost-effectiveness acceptability curves, probabilistic sensitivity analysis on parameter precision and alternate scenario modelling for extrapolation.

The cost analysis will consider the (discounted at 3.5 %) capital expenditure and running and maintenance costs of the fluoridation plant, the Unit of Dental Activity of visits to General Dental Practitioners (costed at standard fees levels), the proportion of General Anaesthetic’s required (costed using the national tariff), and the costs of activities to maintain dental health reported by NHS Business Services Authority for each cohort. For the main cost-effectiveness evaluation, only those costs incurred by NHS and personal and social services are considered. In recognition that WF is a significant cost to other government bodies, we conduct additional analysis where, as a conservative assumption, we plan to divide the program costs by the number of children in the areas covered. This is a conservative assumption, which, if biased, will be biased against finding water fluoridation to be cost-effective. It can be motivated by assuming that the commissioner’s primary focus is on children and/or that the benefits to adults are much smaller or close to zero. In sensitivity analysis, we will examine whether dividing the costs by the total population affects the results.

### Ethics approval and consent

The study has been peer reviewed and approved by an NHS ethics committee and has been approved by the funding organisation NIHR. All participants will provide written informed consent prior to enrolling in the study for themselves (parent/ guardian) and their child.

### Department of Health Disclaimer

The views and opinions expressed therein are those of the authors and do not necessarily reflect those of the NIHR PHR Programme or the Department of Health.

## Discussion

A well-conducted study is required to assess the impact on health and evaluate the value for money (cost effectiveness) of a water fluoridation scheme in the current context of decreasing caries prevalence and population burden. To satisfy the inclusion criteria for a high quality study set out in the York Review a new scheme needs to be implemented and appraised. A unique set of circumstances in Cumbria provides an opportunity to conduct a high quality evaluation of a ‘new’ (reintroduced) water fluoridation scheme, which satisfies the inclusion criteria stipulated by the York systematic review and can address the design issues identified in the MRC report. As a public health intervention the main benefits apply to the whole population receiving the intervention rather than just those participating in the study. There is an overwhelming need for a strong contemporary evidence base for water fluoridation. The changing context of reducing dental disease burden, the consumption of tap water, diet as well as access to evidence based prevention in dental practice have all been recognised by leading bodies (York CRD, MRC) as requiring a robust evaluation of a new water fluoridation scheme [20, 21]. There is a risk that, without such evidence, new water fluoridation schemes may not be introduced – and hence whole populations will be disadvantaged, as they will not receive a highly effective public health measure. Conversely should the intervention prove marginally effective in the current context then there may be a rationale for withdrawal of such interventions elsewhere in England and Wales as populations may be exposed to the risk of fluorosis without any benefit. Society as a whole needs to be informed about decisions regarding the introduction or withdrawal of water fluoridation schemes.

There is therefore a clear benefit to the population in understanding the impact that optimally fluoridated drinking water has on oral health in a contemporary setting. The public need to be informed in order to effectively contribute to debates about the introduction of such schemes in their communities. With the current criticism of the evidence base it is difficult for members of the public to understand the arguments for and against the introduction of such schemes. Additionally the current evidence base is mostly from outside the UK with more recent studies being carried out in places such as Brazil [30]. The NHS, and the wider policy making structures within England Wales, have a similar need for a robust evidence base upon which to develop policy, implement it effectively and understand how to evaluate it.

Water fluoridation has a 70-year history; over 70 % of the population in the USA and over 5 million people in England drink fluoridated water. It is widely advocated as the most cost effective public health measure in reducing dental caries. The headline findings of the York systematic review of water fluoridation that the size of the benefit would be an approximate 15 % increase in the proportion of children with no experience of tooth decay, and a reduction in the mean number of teeth affected by decay of approximately 2.2 teeth. The review also concluded that the benefits of water fluoridation are in addition to the benefits derived from the use of fluoride toothpaste, a conclusion reiterated by a Cochrane systematic review of the effectiveness of fluoride toothpaste [31]. However, the York review also concluded that the evidence base for water fluoridation is limited; most of the studies were conducted at a time before widespread use of fluoride toothpaste and the significant fall we have seen in dental caries prevalence in the UK.

Water fluoridation is believed to have a systemic effect; constant exposure means that fluoride is incorporated into the mineral structure of the teeth as they develop in utero and in the first 5 years of life; and a topical effect once a tooth has erupted by creating an environment at the tooth surface which favours remineralisation. This study will not only aim to assess the impact on developing caries in young children but will also aim to address the economic and quality of life outcomes in regards to water fluoridation.
